# Ferulic acid alleviates sciatica by inhibiting neuroinflammation and promoting nerve repair via the TLR4/NF‐κB pathway

**DOI:** 10.1111/cns.14060

**Published:** 2023-01-04

**Authors:** Di Zhang, Bei Jing, Zhen‐ni Chen, Xin Li, Hui‐mei Shi, Ya‐chun Zheng, Shi‐quan Chang, Li Gao, Guo‐ping Zhao

**Affiliations:** ^1^ College of Traditional Chinese Medicine Jinan University Guangzhou China

**Keywords:** CCI, ferulic acid, GMI‐R1, inflammation, NF‐κB, pain, RSC96, sciatica, TLR4

## Abstract

**Introduction:**

Sciatica causes intense pain. No satisfactory therapeutic drugs exist to treat sciatica. This study aimed to probe the potential mechanism of ferulic acid in sciatica treatment.

**Methods:**

Thirty‐two SD rats were randomly divided into 4 groups: sham operation, chronic constriction injury (CCI), mecobalamin, and ferulic acid. We conducted RNA sequencing, behavioral tests, ELISA, PCR, western blotting, and immunofluorescence analysis. TAK‐242 and JSH23 were administered to RSC96 and GMI‐R1 cells to explore whether ferulic acid can inhibit apoptosis and alleviate inflammation.

**Results:**

RNA sequencing showed that TLR4/NF‐κB pathway is involved in the mechanism of sciatica. CCI induced cold and mechanical hyperalgesia; destroyed the sciatic nerve structure; increased IL‐1β, IL‐6, TNF‐α, IL‐8, and TGF‐β protein levels and IL‐1β, IL‐6, TNF‐α, TGF‐β, TLR4, and IBA‐1 mRNA levels; and decreased IL‐10 and INF‐γ protein levels and IL‐4 mRNA levels. Immunohistochemistry showed that IBA‐1, CD32, IL‐1β, iNOS, nNOS, COX2, and TLR4 expression was increased while S100β and Arg‐1 decreased. CCI increased TLR4, IBA‐1, IL‐1β, iNOS, Myd88, p‐NF‐κB, and p‐p38MAPK protein levels. Treatment with mecobalamin and ferulic acid reversed these trends. Lipopolysaccharide (LPS) induced RSC96 cell apoptosis by reducing Bcl‐2 and Bcl‐xl protein and mRNA levels and increasing Bax and Bad mRNA and IL‐1β, TLR4, Myd88, p‐NF‐κB, and p‐p38MAPK protein levels, while ferulic acid inhibited cell apoptosis by decreasing IL‐1β, TLR4, Myd88, p‐NF‐κB, and p‐p38MAPK levels and increasing Bcl‐2 and Bcl‐xl levels. In GMI‐R1 cells, Ferulic acid attenuated LPS‐induced M1 polarization by decreasing the M1 polarization markers IL‐1β, IL‐6, iNOS, and CD32 and increasing the M2 polarization markers CD206, IL‐4, IL‐10 and Arg‐1. After LPS treatment, IL‐1β, iNOS, TLR4, Myd88, p‐p38MAPK, and p‐NF‐κB levels were obviously increased, and Arg‐1 expression was reduced, while ferulic acid reversed these changes.

**Conclusion:**

Ferulic acid can promote injured sciatic nerve repair by reducing neuronal cell apoptosis and inflammatory infiltration though the TLR4/NF‐κB pathway.

## Graphical abstract

Ferulic acid promotes sciatic nerve repair by inhibiting Schwann cell apoptosis and promoted the transformation of M1 GMI‐R1 microglia to M2 microglia to relieve inflammatory infiltration though the TLR4/NF‐κB pathway.

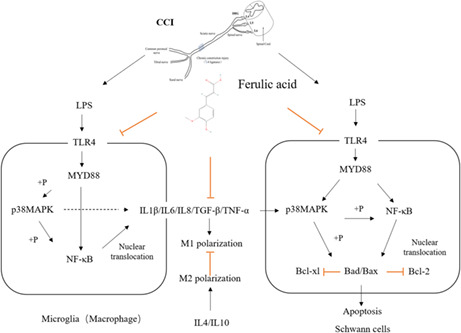

## 1 INTRODUCTION

Sciatica, a common type of neuropathic pain attributed to impingement of the sciatic nerves or injury to the sciatic nerves, is experienced by up to 10% of patients with chronic lower back pain, with a reported lifetime incidence ranging from 10% to 40%^1^ or even up to 70%.^2^ According to the National Institute for Health & Clinical Excellence (NICE) guidelines, noninvasive treatments, including specific exercises, psychological therapy, and nonsteroidal anti‐inflammatory drugs, can be used to treat sciatica.^3^ Gabapentinoids, other antiepileptics, oral corticosteroids or benzodiazepines are not used to manage sciatica because there is no overall evidence of their benefit.^3^ Although nonsteroidal anti‐inflammatory drugs can help relieve sciatica, it is necessary to take into account their potential gastrointestinal, liver and cardio‐renal toxicity.^3^ Moreover, opioids have a risk of addiction in patients.^4^ Thus, it is urgent to seek new therapeutic drugs for the efficacious treatment of sciatica.

Nerve injury and neuroinflammation play vital roles in sciatica. Following injury to the nerve, axonal breakdown is initiated, and the products of degenerated neural tissue stimulate microglia and resident macrophages to secrete chemokines and cytokines, which promote neuroinflammation and axonal breakdown. Activation of microglia leads to the progression of neuropathic pain by interfering with neuronal function.^5^ Inhibiting microglial activation reduces hyperalgesia after nerve damage.^6^ Similarly, Schwann cells undergo dramatic reprogramming from highly quiescent, mature, differentiated myelinating cells to proliferative, prorepair cells after nerve injury and exhibit Wallerian degeneration. In addition, the proliferation and migration of Schwann cells and the inhibition of cell aging and apoptosis can restore the structure of injured peripheral nerves.

TLR4 can induce neuroinflammation and neuralgia. TLR4 is expressed on the cell surface as well as in endosomes, mainly in immune and glial cells.^7^ Myeloid differentiation primary response 88 (MyD88) is the most common adaptor protein that interacts with the intracellular domain of TLR4, which can activate transcription factors such as nuclear factor κ light‐chain enhancer of activated B cells (NF‐κB) and mitogen‐activated protein kinase (MAPK).^8^ Following injury, TLR4 activation on microglia and macrophages contributes to their shift towards an inflammatory phenotype and thus their release of inflammatory factors, including IL‐1β, TNF‐α, and IL‐6.^9^ Intrathecal administration of TLR4 antagonists and siRNA‐mediated suppression of TLR4 signaling prevents activation of the NF‐κB pathway and production of TNF and IL‐1β, which attenuates mechanical allodynia and thermal hyperalgesia in a chronic constriction injury (CCI)‐induced pain model.^10^, ^11^

Ferulic acid exhibits a potential advantage in the treatment of sciatica. Ferulic acid can decrease the levels of oxidative stress, inflammation and apoptosis markers in the sciatic nerves of patients with diabetes.^12^ Ferulic acid exerts a neuroprotective effect against radiation‐induced nerve damage by targeting the NLRP3 inflammasome to enhance learning and memory ability and ameliorate pathological changes in the hippocampal tissues of irradiated mice.^13^ In this study, ferulic acid was found to relieve pain in CCI rats, and we aimed to identify the related mechanisms. The therapeutic effect of ferulic acid on CCI of the sciatic nerve was assessed via behavioral tests, pathological examination, and immunohistochemistry. Next, we investigated the underlying mechanism at the cellular level to provide additional experimental evidence supporting the application of ferulic acid for the treatment of sciatica.

## 2 MATERIALS AND METHODS

### 2.1 Subjects

A total of 32 male Sprague–Dawley rats weighing approximately 150–180 g were acquired from the Experimental Center of Beijing Huafukang Co., Ltd. All animal experiments were conducted in accordance with the guidelines established by the National Academy of Sciences of the National Institutes of Health (NIH) and in accordance with the guidelines of the Animal Ethical Committee of Jinan University. Ethics approval (No. IACUC‐20201223‐07) was obtained on December 23, 2020.

### 2.2 Reagents

Ferulic acid (F103701; 99% purity) was acquired from Shanghai Aladdin Biotechnology Co., Ltd. Mecobalamin (lot number: 1703098) was purchased from Eisai Pharmaceutical Co., Ltd. PageRμLer Prestained Protein Ladder and Marker (P12083) was acquired from Shanghai Bioscience Technology Co., Ltd. RIPA buffer (WB‐0071) was purchased from Beijing Dingguo Biological Co., Ltd. RNAiso Plus (9108) was acquired from Takara Biomedical Technology Co., Ltd. SYBR Green Premix qPCR, an RT–PCR Kit, and RNase‐free water (AG11701, AG11602, AG11012) were obtained from Accurate Biotechnology Co., Ltd. IL‐1β (MM‐0047R1), IL‐6 (MM‐0190R1), TNF‐α (MM‐0180R1), IL‐8 (MM‐0175R1), IL‐10 (MM‐195R1), TGF‐β (MM‐20594R1), and IFN‐γ (MM‐0198R1) antibodies were purchased from Jiangsu Meimian Industrial Co., Ltd. Lipopolysaccharide (LPS, L2880), JSH‐23 (M134534, an NF‐κB inhibitor) and TAK‐242 (S80562, a TLR4 inhibitor) were acquired from Guangzhou Yiyou Biotechnology Biological Co., Ltd. The following antibodies were used: anti‐nNOS (CPA5524, Cohesion), anti‐CD32 (40700, SAB), anti‐CD206 (DF4149, Affinity), anti‐COX2 (33345, SAB), anti‐IBA‐1 (A5595, Bimake), anti‐IL‐1β (511369S, CST), anti‐iNOS (2982S, CST), anti‐TLR4 (505258, Zen Bio), anti‐Myd88 (4283S, CST), anti‐NF‐κB (8242S, CST), anti‐p‐NF‐κB (3033, CST), anti‐p38MAPK (8690S, CST), anti‐p‐p38MAPK (4511S, CST), anti‐Bcl‐2 (ab59348, Abcam), anti‐Bcl‐xl (2764S, CST), anti‐S100β (GB13359, Servicebio), anti‐β‐actin (4970S, CST), and anti‐Arg‐1 (93668S, CST). All antibodies were diluted 1:1000. A phosphatase inhibitor cocktail was purchased from Servicebio Biological Technology. An Annexin V APC Apoptosis Detection Kit I (62700‐80) was purchased from Guangzhou Squirrel Biological Co., Ltd. An MRC1 polyclonal antibody (CD206) (PA5‐114370) was purchased from Thermo Fisher Scientific Co., Ltd. A PE‐conjugated anti‐mouse CD16/32 (156605) antibody was purchased from BioLegend Biotechnology Co., Ltd. Fixation buffer (420801) and intracellular staining perm wash buffer (421002) were purchased from Dakewe Biotech Co., Ltd. (Shenzhen, China). GMI‐R1 cells (microglia) were acquired from Huatuo Biotechnology Biological Co., Ltd. Schwann cells (RSC96 cells) were acquired from the Shanghai Institute of Cell Biology (GNR6).

### 2.3 Sciatica model

The CCI model (sciatica model) was constructed as described in previous studies.^14^, ^15^, ^16^, ^17^, ^18^, ^19^ Before the mice were anesthetized with pentobarbital sodium (3%; 40 mg/kg) and fixed to the operation table, the rats were fasted for 12 h. After exposing the sciatic nerve under a microscope, the right sciatic nerve was tied 4 times with 4.0 sutures at intervals of approximately 1 mm. At this point, we observed a small twitch in the operated hind limb. The rats in the sham group did not undergo nerve ligation. Finally, gentamicin (10 mg/ml, i.m.) was injected.

### 2.4 Treatment programs

Thirty‐two rats were randomly divided into four groups: the sham operation group, the CCI group, the mecobalamin group, and the ferulic acid group. The rats in the sham operation group and the CCI group were given saline (0.9%, 12 ml/kg), the rats in the mecobalamin group received a gavage of mecobalamin (20 mg/kg), and the rats in the ferulic acid group received ferulic acid (100 mg/kg) by gavage.^20^ The drugs were administered for 21 days.

### 2.5 Behavioral tests

To assess cold hyperalgesia, we chose the acetone experiment. A total of 100 μl of acetone was dropped on the plantar surface of the right paw. Next, we noted the total number of times that the rat lifted or clutched its right hind paw within 120 s. The von Frey test was used to evaluate mechanical hyperalgesia (50% mechanical withdrawal threshold [MWT]). The hind paw was stimulated 10 times with each filament (2.0–26.0 g) beginning with the 2‐g filament, and paw lifting was considered a positive response. If we detected a positive response, we calculated the pain threshold (50% g threshold = 10^[Xf+kδ]/10,000^). We assessed hyperalgesia of the right paw on the 1st, 4th, 7th, 14th, and 21st days after surgery. All tests were repeated three times at 10‐min intervals for each paw, and the mean was calculated.

### 2.6 RNA sequencing

The sciatic nerves of rats in the sham operation group and CCI group were collected 21 days postinjury. Total RNA was isolated using RNAiso Plus. Subsequently, the concentration and quality of the total RNA were assessed using a Nano Drop and Agilent 2100 bioanalyzer (Thermo Fisher Scientific). After the mRNA was purified with oligo(dT)‐attached magnetic beads, it was fragmented into small pieces with fragment buffer at the appropriate temperature. Then, first‐strand cDNA was generated using random hexamer‐primed reverse transcription, followed by second‐strand cDNA synthesis. Afterwards, A‐Tailing Mix and RNA Index Adapters were added by incubation for end repair. The cDNA fragments obtained in the previous step were amplified by PCR, and the products were purified by Ampure XP Beads and then dissolved in EB solution. The products were validated with an Agilent Technologies 2100 bioanalyzer for quality control. The double‐stranded PCR products obtained in the previous step were heated, denatured and circularized by the splint oligo sequence to obtain the final library. Single‐strand circular DNA (ssCir DNA) was formatted as the final library. The final library was amplified with phi29 to make DNA nanoballs (DNBs), which had more than 300 copies of a single molecule. DNBs were loaded into the patterned nanoarray, and paired‐end 150‐base reads were generated on the DNBSEQ‐T7 platform by Tsingke Biotechnology Co., Ltd. The raw reads were filtered using the Trim Galore method (https://ccb.jhu.edu/software/hisat2/index.shtml) to obtain clean reads for subsequent analysis and to ensure the quality of the information analysis. The clean reads obtained after filtering were compared with the reference database annotations (the Rno6 version of the rat genome was selected) using HISAT2 software (https://ccb.jhu.edu/software/hisat2/index.shtml). Differentially expressed genes (DEGs) were screened using Sangerbox, and two criteria were used for screening the DEGs: a false discovery rate (FDR) ≤0.05 and |Log2‐fold change (FC) | ≥ 1. Then, we used DAVID to conduct KEGG enrichment analysis.

### 2.7 H&E staining, immunohistochemistry, and ELISA

Liver, kidney, and sciatic nerve tissues were fixed in 4% paraformaldehyde and then sliced at a thickness of 3 μm for H&E staining and 9 μm for immunohistochemistry. The sections were deparaffinized, washed with PBS for 5 min, stained with H&E, and washed with water. The sciatic nerve sections were incubated in sodium citrate antigen repair solution (1:1000 dilution; pH = 6). Next, the sections were incubated with primary antibodies diluted to 1:200, followed by the corresponding secondary antibody. The stained sections were viewed under an Olympus fluorescence microscope (BX53). We calculated the IOD/area ratio using Image‐Pro Plus software (Media Cybernetics, Inc.) and conducted statistical analysis.

To measure the concentrations of serum inflammatory factors, we collected 8 ml of blood from the abdominal aorta. The blood samples were centrifuged at 4032 *g* for 15 min at 4°C, and 800 μl of the supernatant was retained for measurement of inflammatory factor levels. The remaining steps were performed according to the manufacturer's instructions. We used a microplate reader (Bio Tek Instruments, Inc.) to measure the optical density.

### 2.8 Cell viability and cytotoxicity assays

The viability of GMI‐R1 cells and RSC96 cells was determined by the CCK‐8 assay. According to the results of the cell viability assay, 10 μg/ml, 10 μM, 10 μM, and 2 μM were selected as the concentrations of LPS, TAK‐242, JSH23, and ferulic acid, respectively.^15^, ^20^

### 2.9 Grouping for the cell experiment

Cells were divided into the control group, LPS group (10 μg/ml LPS), TAK‐242 group (10 μg/ml LPS + 10 μM TAK‐242), JSH23 group (10 μg/ml LPS + 10 μM JSH23), LPS + ferulic acid group (10 μg/ml LPS + 2 μM ferulic acid), and ferulic acid group (2 μM ferulic acid). GMI‐R1 cells and RSC96 cells were cultured in 6‐well plates for 24 h. Next, the medium was discarded, and the cells were washed with PBS. Drugs and LPS were added to the medium at the same time, and then the cells were cultured for 24 h.

### 2.10 Flow cytometry analysis

We collected and washed GMI‐R1 cells and RSC96 cells. We first measured the percentages of M1 and M2 microglia among LPS‐treated GMI‐R1 cells. The membrane protein CD32 was detected by direct staining. The cells were fixed using fixation buffer, permeabilized twice with intracellular staining perm wash buffer, and incubated with a PE‐conjugated monoclonal mouse CD16/32 antibody in the dark for 30 min. Then, the cells were fixed using fixation buffer, blocked and incubated with a polyclonal MRC1 antibody, followed by DyLight 638‐conjugated goat anti‐rabbit IgG for 30 min in the dark. The cells were washed twice with PBS and resuspended in 500 μl of PBS. Annexin V‐APC and PI staining were conducted to evaluate the rate of RSC96 cell death. After treatment, the cells were harvested with trypsin and washed with PBS. Then, the cells were incubated in binding buffer and double stained with Annexin V‐APC and PI in the dark for 20 min at 4°C. The light scattering properties of each sample (10^5^ cells) were analyzed using a flow cytometer (CytoFLEX, Beckman Coulter) equipped with FlowJo software.

### 2.11 Quantitative real‐time PCR

Total RNA was harvested using RNAiso Plus and synthesized into cDNA with an RT–PCR kit according to the manufacturer's instructions. The relative mRNA expression was calculated by the 2^−ΔΔCq^ method after normalization to the level of β‐actin expression.^21^ The Applied Biosystems 7900 real‐time PCR (qPCR) system, SYBR® Green Premix qPCR, and primers, which are shown in Table 1, were used for quantitative real‐time PCR.

**TABLE 1:** Primer sequences

Gene	Forward primer (5′–>3′)	Reverse primer (5′–>3′)
Arg‐1	CAGTATTCACCCCGGCTA	CCTCTGGTGTCTTCCCAA
Bad	GCAGCCAATAACAGTCAT	CTAAGCTCCTCCTCCATC
Bax	CTGGACAACAACATGGAG	AAGTAGAAAAGGGCAACC
Bcl‐2	CAGGCTGGAAGGAGAAGAT	CGGGAGAACAGGGTATGA
Bcl‐xl	TAGGTGGTCATTCAGGTAGG	GTGGAAAGCGTAGACAAGG
IBA‐1	ATCAACAAGCACTTCCTC	ATATCTCCATTGCCATTC
IL‐10	AGGGTTACTTGGGTTGCC	GGGTCTTCAGCTTCTCTCC
IL‐1β	AGGAGAGACAAGCAACGACA	CTTTTCCATCTTCTTCTTTGGGTAT
IL‐4	CAAGGAACACCACGGAGAA	AGCACGGAGGTACATCACG
IL‐6	AGTTGCCTTCTTGGGACTGATGT	GGTCTGTTGTGGGTGGTATCCTC
TGF‐β	ACAGGGCTTTCGCTTCAGT	AGGTCACCTCGACGTTTGG
TLR4	ATCAGTGTATCGGTGGTCAGT	AGCCAGCAATAAGTATCAGGT
TNF‐α	GCGTGTTCATCCGTTCTCTACC	TACTTCAGCGTCTCGTGTGTTTCT
β‐Actin	CCTAGACTTCGAGCAAGAGA	GGAAGGAAGGCTGGAAGA

### 2.12 Western blot analysis

The levels of TLR4, IBA‐1, Arg‐1, IL‐1β, iNOS, Myd88, NF‐κB, p‐NF‐κB, p38MAPK, p‐p38MAPK, Bcl‐2, Bcl‐xl, and β‐actin were measured by western blotting. Total protein was extracted as previously described (Zhang et al., 2021b). We acquired the membrane fraction with a cell membrane protein and cytoplasmic protein extraction kit. The proteins (10 μg) were separated by 12% SDS‐PAGE and transferred onto PVDF membranes, which were blocked with 5% skimmed milk powder for 1 h, incubated with primary antibody (1:1000) overnight at 4°C for 24 h and incubated with secondary antibody (1:30,000) for 1 h. Finally, a ChemiDoc XRS imager was used to visualize the bands. Each experiment was conducted in triplicate.

### 2.13 Statistical analyses

We used GraphPad Prism 8 to generate all graphs and to perform all analyses. The values are expressed as the mean ± standard deviation. Formal tests for normality were used to assess data distributions. All data were subjected to tests for normality. The behavior data were analyzed by repeated‐measures ANOVA. The other data were analyzed by one‐way ANOVA. Tukey's multiple comparisons test was performed after ANOVA. The results with a *p* value of <0.05 were considered statistically significant.

### 2.14 Signaling pathways

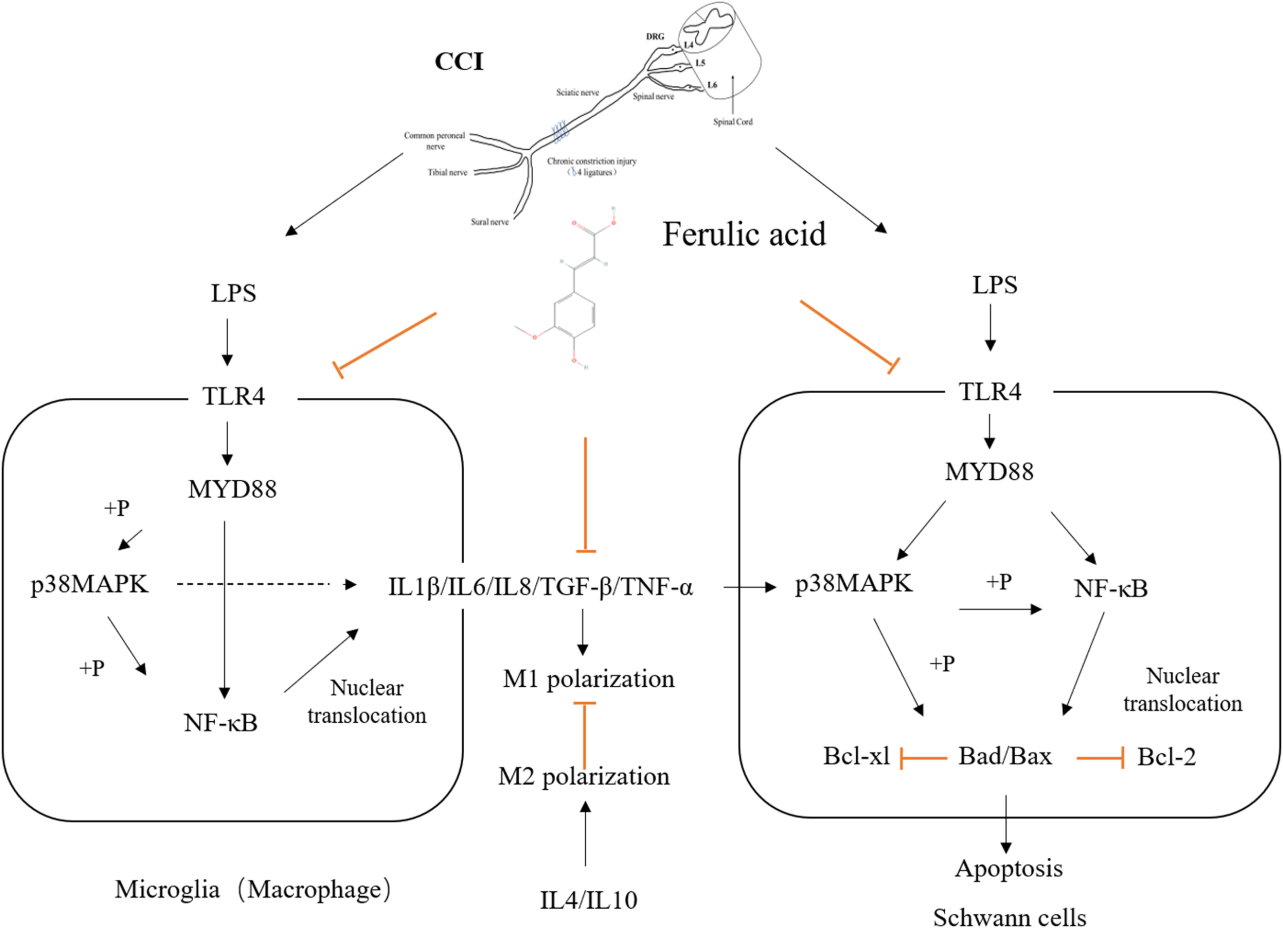

## 3 RESULTS

### 3.1 Behavioral tests

**FIGURE 1:**
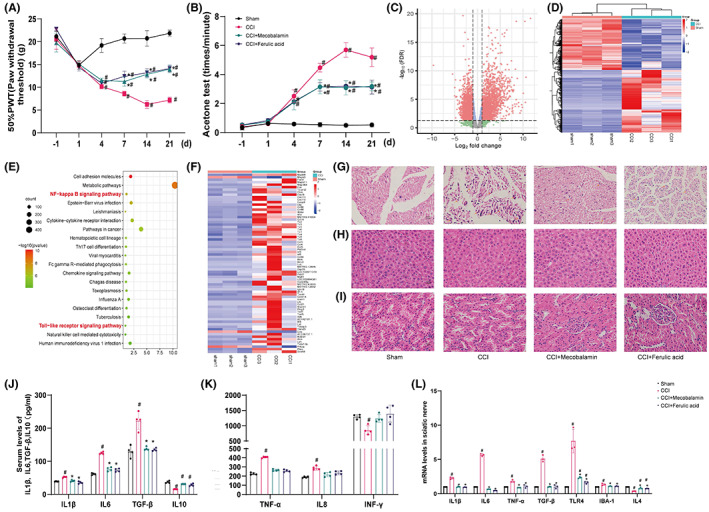
Ferulic acid attenuated CCI‐induced neuropathic pain. (A, B) Behavioral results (A, von Frey test; B, acetone experiment). (C) RNA sequencing of tissues from the sham operation group and CCI group. (D) Heatmap of the differentially expressed genes between the sham operation group and the CCI group. (E) KEGG pathway enrichment analysis. (F) Heatmap of the differentially expressed genes associated with both the “NF−kappa B signalling pathway” and the “Toll‐like receptor signalling pathway.” (G–I) H&E staining of the sciatic nerve, liver, and kidney. (J, K) Levels of serum inflammatory factors. (L) mRNA levels in the sciatic nerve. ^#^Compared with the sham operation group; *compared with the CCI group; *p* < 0.05.

After CCI, rats exhibited cold and mechanical hyperalgesia from the 4th day to the 21st day (Figure 1A,B; *p* < 0.05). Treatment with ferulic acid and mecobalamin (positive control drug) relieved neuropathic pain but did not normalize sensitivity from the 4th day to the 21st day (*p* < 0.05). The analgesic effects of ferulic acid were not different from those of mecobalamin (*p* > 0.05). These results showed a lack of full functional recovery of the injured sciatic nerves.

### 3.2 Analysis of DEGs and KEGG pathway analysis

In the present study, DEGs were identified by comparing gene expression in the sciatic nerve between the sham operation group and the CCI group. A total of 14,622 genes were found, and 3777 genes with an FDR ≤0.05 and |Log2(FC)| ≥ 1 (Figure 1C,D) were selected as DEGs. A heatmap (Figure 1D) was used to visualize the expression of the differentially expressed genes in each sample. Then, we performed KEGG pathway analysis of the 3777 DEGs via the DAVID database (Figure 1E; *p* < 0.05). KEGG pathway analysis (Figure 1E) revealed that the relationship between the “NF−kappa B signalling pathway” and the “Toll‐like receptor signalling pathway” was quite close, and the two pathways ranked in the top 20. The “NF‐kappa B signaling pathway” is ranked in the top 3, and the “TLR4/NF‐κB” pathway is also involved. The “NF‐kappa B signaling pathway” is involved in the “Toll‐like receptor signaling pathway.” We chose the TLR4/NF‐κB pathway to conduct experimental verification. The differentially expressed genes associated with these two pathways are shown in Figure 1E,F.

### 3.3 H&E staining

The structures of the liver, kidney, and sciatic nerve were observed via H&E staining. The neural structure of the sciatic nerve (Figure 1G) was normal in the sham operation group but was destroyed after CCI. Ferulic acid and mecobalamin helped restore nerve structure. Liver and kidney structures (Figure 1H,I) were normal in all groups, which indicated that neither the drugs nor CCI had negative effects on the livers and kidneys of the rats.

### 3.4 Serum inflammatory factor and mRNA levels in the sciatic nerve

The levels of IL‐1β, IL‐6, TNF‐α, IL‐8, IL‐10, TNF‐α, TGF‐β, and INF‐γ in the serum were measured via ELISA. The levels of IL‐1β, IL‐6, IL‐8, TNF‐α, and TGF‐β were significantly increased, and the expression levels of IL‐10 and INF‐γ were decreased after CCI (Figure 1J,K; *p* < 0.05). After treatments, ferulic acid and mecobalamin decreased the levels of IL‐1β, IL‐6, IL‐8, TNF‐α, and TGF‐β to nearly normal levels and increased the level of INF‐γ to normal levels (*p* < 0.05), but they could not restore the expression of IL‐10 to normal levels. To assess neuroinflammation in the sciatic nerve, we measured the mRNA levels of IL‐1β, IL‐6, IL‐4, TNF‐α, TGF‐β, TLR4, and IBA‐1 in the sciatic nerve and observed that the mRNA levels of IL‐1β, IL‐6, TNF‐α, TGF‐β, TLR4, and IBA‐1 were significantly increased after CCI, while the mRNA level of IL‐4 was reduced (Figure 1L; *p* < 0.05). Ferulic acid and mecobalamin lowered the levels of IL‐1β, IL‐6, TNF‐α, TLR4, and IBA‐1 to normal levels, and ferulic acid decreased the level of TGF‐β to normal levels (*p* < 0.05). However, they could not normalize the level of IL‐4.

### 3.5 Immunohistochemical staining of the sciatic nerve

**FIGURE 2:**
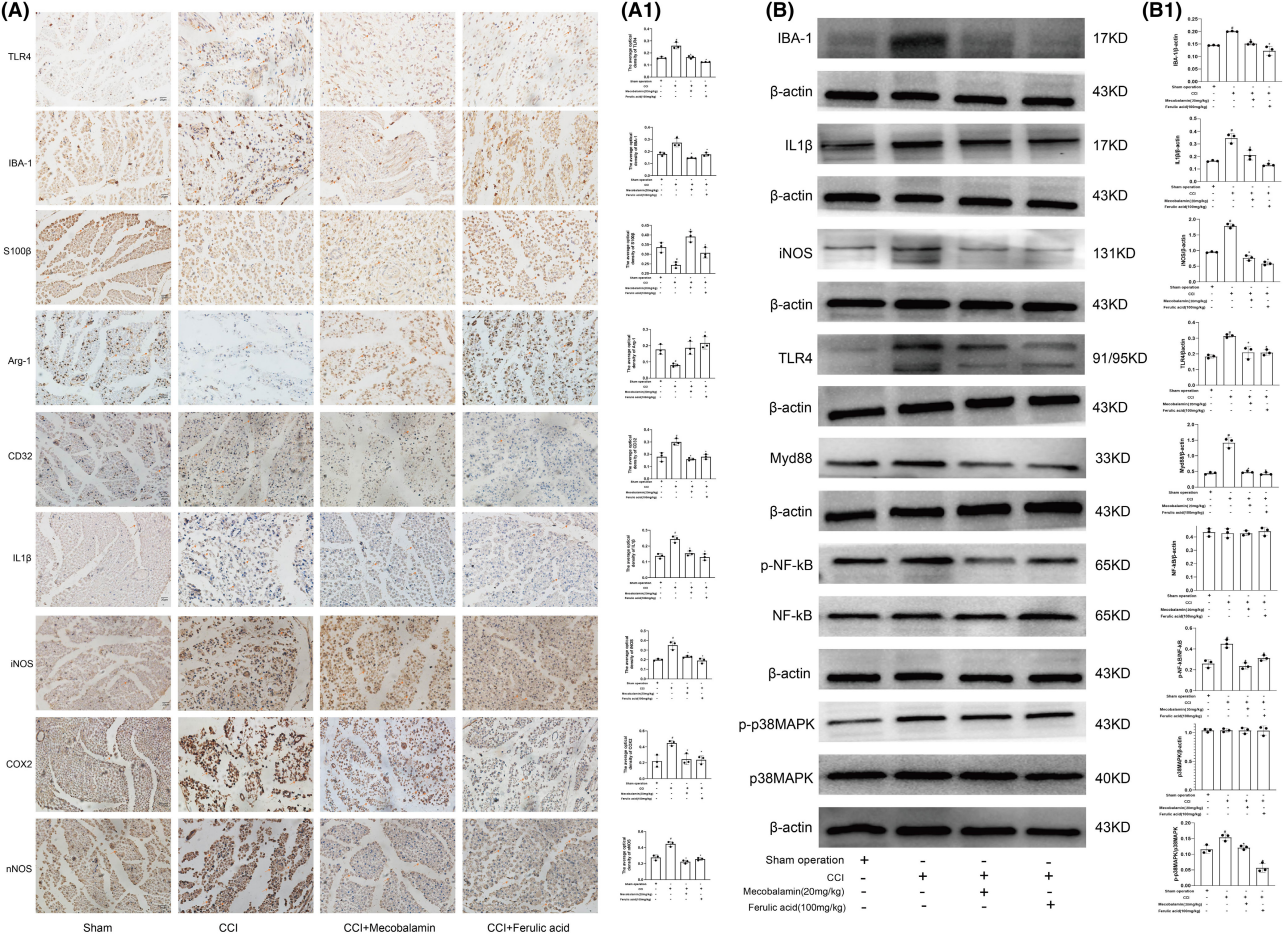
Immunohistochemical analysis and effects of ferulic acid on the TLR4/NF‐κB pathway in the sciatic nerve. (A) Immunohistochemical staining of IBA‐1, IL‐1β, CD32, iNOS, nNOS, COX2, Arg‐1, S100β and TLR4. (A1) Statistical analysis. (B) Western blots. (B1) Statistical analysis. ^#^Compared with the sham operation group; * compared with the CCI group; *p* < 0.05.

IBA‐1 (a microglial and macrophage marker), M1 polarization markers (IL‐1β, CD32, and iNOS), nNOS, COX2, and TLR4 were expressed at low levels in the normal sciatic nerve in the sham operation group but were expressed at higher levels after CCI (Figure 2A; *p* < 0.05). The levels of IBA‐1, IL‐1β, CD32, iNOS, nNOS, COX2, and TLR4 in the ferulic acid and mecobalamin groups were lower than those in the CCI group (*p* < 0.05). S100β (a Schwann cell marker) and Arg‐1 (an M2 polarization marker) were expressed at higher levels in the sham operation group and at lower levels in the CCI group (Figure 2A; *p* < 0.05). Ferulic acid and mecobalamin increased the expression of S100β and Arg‐1 (*p* < 0.05).

### 3.6 Effects of ferulic acid on the TLR4/NF‐κB pathway

After CCI, the levels of IBA‐1, IL‐1β, iNOS, TLR4, Myd88, p‐NF‐κB, and p‐p38MAPK were obviously increased (Figure 2B; *p* < 0.05). Ferulic acid and mecobalamin decreased the levels of these proteins. The levels of p38MAPK and NF‐κB were nearly equal among the groups (*p* < 0.05). In addition, ferulic acid and mecobalamin restored the levels of IBA‐1, IL‐1β, iNOS, TLR4, Myd88, p‐NF‐κB, and p‐p38MAPK to nearly normal levels (*p* < 0.05).

### 3.7 Ferulic acid alleviated LPS‐induced apoptosis via the TLR4/NF‐κB pathway in RSC96 cells

**FIGURE 3:**
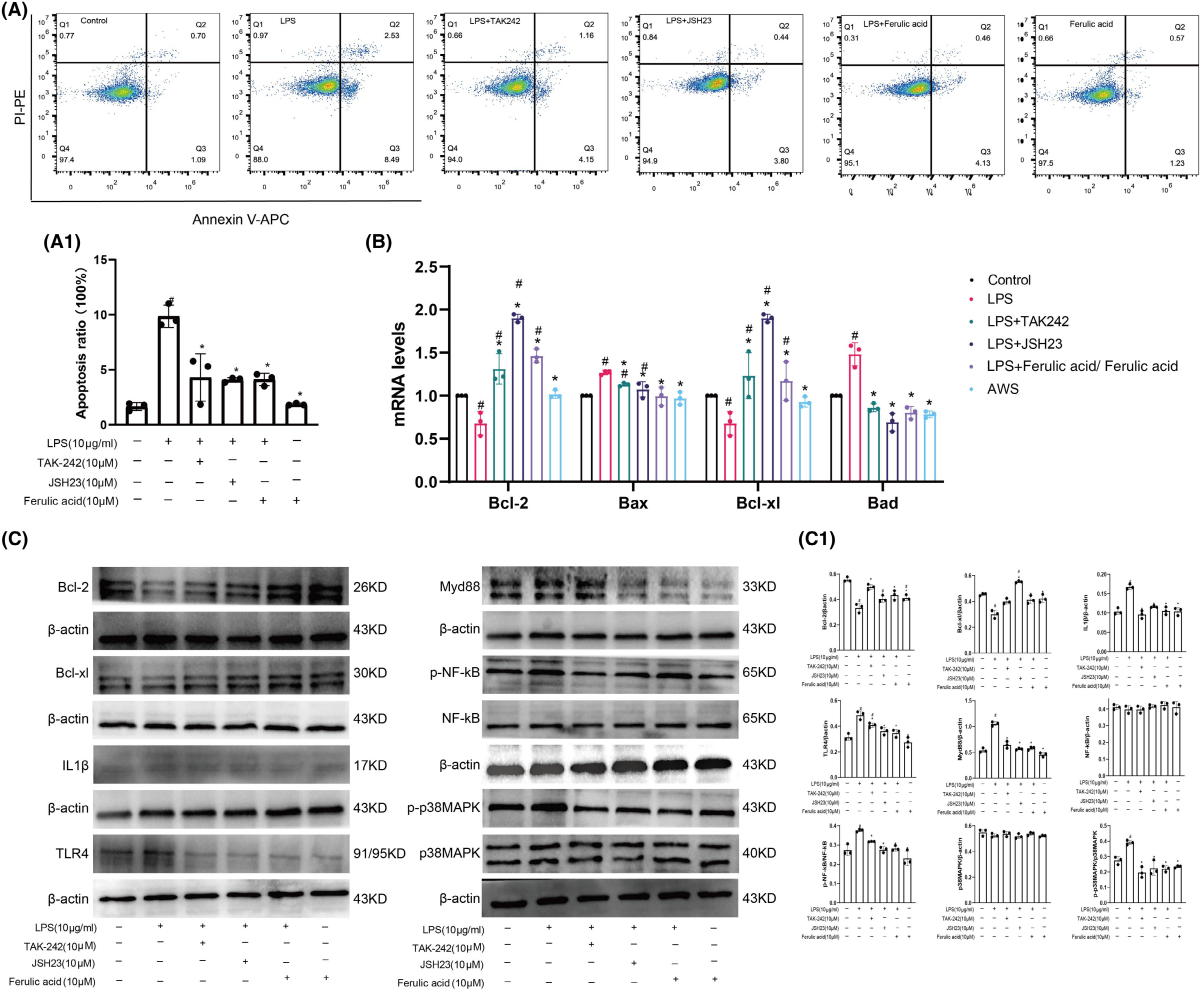
Ferulic acid alleviated LPS‐induced apoptosis via the TLR4/NF‐κB pathway in RSC96 cells. (A) Flow cytometry. (A1) Statistical analysis. (B) mRNA levels of Bax, Bcl‐2, Bad, and Bcl‐xl. (C) Western blots. (C1) Statistical analysis. ^#^Compared with the sham operation group; * compared with the CCI group; *p* < 0.05.

Flow cytometry indicated that LPS induced RSC96 cell apoptosis (Figure 3A; *p* < 0.05). PCR showed that LPS increased the mRNA expression levels of Bax and Bad and decreased the mRNA levels of Bcl‐2 and Bcl‐xl (Figure 3B; *p* < 0.05). Ferulic acid, TAK‐242 or JSH23 inhibited LPS‐induced cell apoptosis (*p* < 0.05), but ferulic acid alone did not affect Schwann cell viability (Figure 3A). In addition, ferulic acid combined with TAK‐242 or JSH23 lowered the mRNA level of Bad to normal levels (*p* < 0.05), but it could not normalize the level of Bax or increase the levels of Bcl‐2 and Bcl‐xl to normal levels (Figure 3B; *p* < 0.05).

LPS increased the protein levels of IL‐1β, TLR4, Myd88, p‐NF‐κB, and p‐p38MAPK and reduced the expression levels of Bcl‐2 and Bcl‐xl, and the levels of p38MAPK and NF‐κB were almost normal in all groups (Figure 3C; *p* < 0.05). After treatment with ferulic acid, TAK‐242 or JSH23, the expression of IL‐1β, TLR4, Myd88, p‐NF‐κB, and p‐p38MAPK was restored almost to normal levels (Figure 3C; *p* < 0.05). These results indicated that TAK‐242, JSH23, and ferulic acid alleviated LPS‐induced RSC96 cell apoptosis.

### 3.8 Ferulic acid promoted the transformation of M1 GMI‐R1 microglia to M2 microglia following LPS treatment via the TLR4/NF‐κB pathway

**FIGURE 4:**
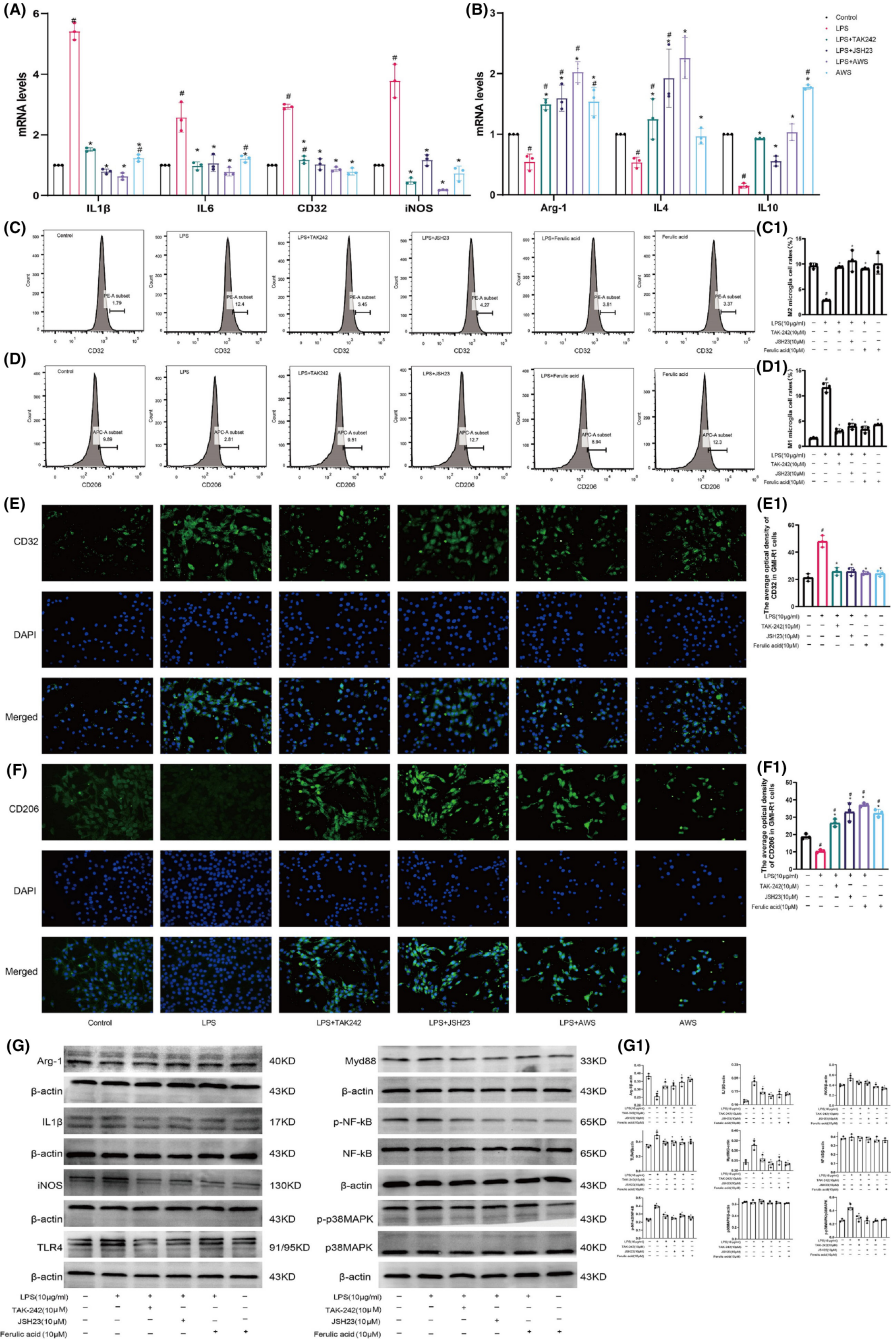
Ferulic acid promoted the transformation of M1 microglia to M2 microglia via the TLR4/NF‐κB pathway. (A) M1 microglial marker levels. (B) M2 microglial marker levels. (C) The proportion of M1 microglia (CD32) was determined via flow cytometry. (C1) Statistical analysis. (D) The proportion of M2 microglia (CD206) was determined via flow cytometry. (D1) Statistical analysis. (E) Immunofluorescence of CD32. (E1) Statistical analysis. (F) Immunofluorescence of CD206. (F1) Statistical analysis. (G) Western blots. (G1) Statistical analysis. ^#^Compared with the sham operation group; * compared with the CCI group; *p* < 0.05.

To explore whether ferulic acid has anti‐inflammatory effects, we conducted PCR and flow cytometry to assess whether ferulic acid regulates the levels of inflammatory cytokines. Figure 4 shows that the mRNA levels of the M1 microglia‐related proinflammatory cytokines IL‐1β, IL‐6, iNOS, and CD32 were increased after LPS treatment but decreased after treatment with TAK‐242, JSH23, and ferulic acid (Figure 4A; *p* < 0.05). The levels of M2 microglia‐related anti‐inflammatory cytokines (IL‐4, IL‐10 and Arg‐1) were decreased by LPS stimulation but increased after treatment with TAK‐242, JSH23, and ferulic acid (Figure 4B; *p* < 0.05). Flow cytometry and immunofluorescence showed that LPS induced the M1 polarization of microglia (Figure 4C,E; *p* < 0.05); TAK‐242, JSH23, and ferulic acid had a repressive effect on the LPS‐induced M1 polarization of microglia; and ferulic acid alone had no effect on microglia (Figure 4D,F; *p* < 0.05). LPS reduced the proportion of CD206‐positive microglia (Figure 4D,F; *p* < 0.05); TAK‐242, JSH23, and ferulic acid increased the proportion of M2 microglia; and ferulic acid alone increased the proportion of M2 microglia (Figure 4D1,F1). These results indicated that TAK‐242, JSH23, and ferulic acid promoted the transformation of M1 microglia to M2 microglia.

The protein levels of iNOS, IL‐1β, TLR4, Myd88, p‐NF‐κB and p‐p38MAPK were obviously increased, while the expression of Arg‐1 was reduced (Figure 4G; *p* < 0.05). TAK‐242, JSH23, and ferulic acid reduced the expression levels of iNOS, IL‐1β, TLR4, Myd88, p‐NF‐κB and p‐p38MAPK and increased the level of Arg‐1. The levels of p38MAPK and NF‐κB were almost equivalent among the groups. Ferulic acid reduced the levels of iNOS, IL‐1β, TLR4, Myd88, p‐NF‐κB and p‐p38MAPK to approximately normal levels (Figure 4G; *p* < 0.05). These results indicated that ferulic acid promoted the transformation of M1 microglia to M2 microglia via the TLR4/NF‐κB signaling pathway.

## 4 DISCUSSION

In this study, the results of rat and cell experiments provide some convincing evidence that ferulic acid can treat sciatica. The major findings are as follows: (1) ferulic acid effectively alleviated neuroinflammation and promoted sciatic nerve repair; (2) the development of sciatica may be closely related to the “NF−kappa B signalling pathway” and the “Toll‐like receptor signalling pathway”; (3) CCI led to sciatic nerve injury and neuroinflammation with higher expression levels of TLR4, Myd88 and p‐NF‐κB in the sciatic nerve; (4) ferulic acid suppressed Schwann cell apoptosis induced by LPS in an inflammatory environment via the TLR4/NF‐κB pathway; and (5) ferulic acid promoted the transformation of M1 microglia to M2 microglia to suppress neuroinflammation via the TLR4/NF‐κB pathway.

We conducted RNA sequencing of sciatic nerves from the sham operation group and the CCI group. Given that the “NF−kappa B signalling pathway” ranked in the top 3 and the “Toll‐like receptor signalling pathway” ranked in the top 20, these two pathways are closely related to the mechanism of sciatica. Based on the heatmap (Figure 1F), the TLR4/NF‐κB pathway was selected for follow‐up research. The TLR4/NF‐κB pathway is closely related to apoptosis and inflammation.^22^ Serum ELISA and PCR of the sciatic nerve showed that nerve injury can increase the levels of inflammatory factors, including IL‐1β, IL‐6, TNF‐α, and IL‐8, and western blotting showed that CCI can lead to higher protein levels of TLR4, MyD88, IL‐1β, and p‐NF‐κB, which verified the RNA sequencing results.

We evaluated the therapeutic effect of ferulic acid on the CCI model via the von Frey test and acetone experiment. Ferulic acid relieved cold and mechanical hyperalgesia, and H&E staining showed that ferulic acid promoted nerve repair. The results of ELISA and PCR demonstrated that ferulic acid decreased the levels of inflammatory factors (IL‐1β, IL‐6, IL‐8, TGF‐β, and TNF‐α) and increased the levels of anti‐inflammatory factors (IL‐4, IL‐10, and INF‐γ). Western blotting and immunohistochemistry indicated that ferulic acid decreased the expression levels of TLR4, IBA‐1, iNOS, IL‐1β, Myd88, p‐p38MAPK, and p‐NF‐κB. These results indicated that ferulic acid may reduce inflammatory factor levels and promote nerve repair. Therefore, we verified the anti‐inflammatory mechanism of ferulic acid in GMI‐R1 cells and the mechanism by which it inhibits apoptosis in RSC96 cells.

S100β is a marker of Schwann cells, which secrete neurotrophic factors and provide structural support and guidance to promote nerve regeneration.^23^ The autologous transplantation of Schwann cells can promote human peripheral nerve repair in 7.5‐cm and 5‐cm sciatic nerve injuries.^24^ After CCI, S100β distribution was altered, and CCI reduced the expression of S100β, indicating that the normal nerve structure was destroyed. However, ferulic acid increased the levels of S100β and promoted nerve repair. A TLR4 inhibitor (TAK‐242) and an NF‐kB inhibitor (JSH23) suppressed LPS‐induced apoptosis by increasing the mRNA and protein levels of Bcl‐2 and Bcl‐xl and decreasing the mRNA levels of Bax and Bad in Schwann cells (RSC96). Ferulic acid attenuated LPS‐induced Schwann cell apoptosis by decreasing the levels of TLR4, p‐NF‐κB, p‐p38MAPK, IL‐1β, Bcl‐2, and Bcl‐xl; increasing the mRNA levels of Bcl‐2 and Bcl‐xl; and decreasing the mRNA levels of Bax and Bad. These results indicated that ferulic acid reduces Schwann cell apoptosis via the TLR4/NF‐κB pathway.

IBA‐1 is a marker of activated microglia and macrophage. Activation of microglia and macrophage induces neuroinflammation.^25^ Macrophages in the sciatic nerve had some characteristics of microglia.^26^ Acute injury of the sciatic nerve led to a rapid infiltration of circulating monocytes, and the monocytes quickly adapted a macrophage phenotype.^27^ The GMI‐R1 cell (microglia) was used to conduct the vitro experiments in this study. Immunohistochemical analysis of the sciatic nerve showed that CCI led to the activation of macrophage or microglia and that ferulic acid suppressed these activations. ELISA showed that CCI increased the expression levels of IL‐1β, IL‐6, TNF‐α, IL‐8, and TGF‐β and decreased the levels of IL‐4 and IL‐10 and that ferulic acid reversed these trends. PCR showed that CCI increased the mRNA levels of IL‐1β, IL‐6, TNF‐α, TLR4, IBA‐1, and TGF‐β and decreased the levels of IL‐4 and that ferulic acid reversed these trends. In cells experiments, M1 microglia had increased expression of several proteins and cytokines, including iNOS, CD32, IL‐1β, IL‐6, while M2 microglia had increased expression of several proteins and cytokines, such as Arg‐1, CD206, IL‐4, and IL‐10.^28^ LPS increased the proportion of M1 microglia, while ferulic acid, TAK‐242, and JSH23 increased the proportion of M2 microglia, including increasing the expression of Arg‐1, IL‐4, and IL‐10 and decreasing the levels of IL‐1β, IL‐6, CD32, and iNOS. In addition, CCI increased the protein levels of TLR4, Myd88, IL‐1β, p‐p38MAPK, and p‐NF‐kB, and ferulic acid decreased the levels of these proteins in rats. Similarly, LPS increased the expression levels of TLR4, MyD88, IL‐1β, p‐p38 MAPK, and p‐NF‐κB, while TAK‐242, JSH23, and ferulic acid decreased the expression levels of these proteins. These results indicated that ferulic acid can inhibit neuroinflammation via the TLR4/NF‐κB pathway.

Since ferulic acid can alleviate sciatica by inhibiting neuroinflammation, promoting sciatic nerve repair and exerting an analgesic effect via the TLR4/NF‐κB pathway, ferulic acid might be developed as a novel therapeutic drug.

However, there were limitations to the present study; specifically, the number of samples for RNA sequencing was not adequate. Three samples in each group exhibited individual differences that could not be avoided. In addition, primary cells should be used for cell experiments in the future. For cell experiments, it is better to add the experiments of macrophages in vitro.

## 5 CONCLUSION

Ferulic acid can alleviate sciatica in CCI rats and inhibit neuroinflammation, promote sciatic nerve repair and exert an analgesic effect via the TLR4/NF‐κB pathway.

## AUTHOR CONTRIBUTIONS

Di Zhang, Bei Jing, Zhenni Chen, and Guoping Zhao contributed substantially to the experimental design, data analysis and experimental procedures. Huimei Shi, Xin Li, Shiquian Chang, Zhenni Chen, Li Gao, and Yachun Zheng assisted with the English writing and partial experiments. We thank Yixuan Li for her valuable comments on the statistical analysis and English writing. Guoping Zhao is the corresponding author. All data were generated in‐house, and no paper mill was used. All authors agree to be accountable for all aspects of the work, ensuring its integrity and accuracy.

## CONFLICT OF INTEREST

The authors declare no conflicts of interest.

## Data Availability

The data used to support the findings of this study are available from the corresponding author upon request.
